# Synergistic effects of temperature and salt stress on seed germination and the antioxidant enzyme system of okra

**DOI:** 10.3389/fpls.2026.1822950

**Published:** 2026-07-14

**Authors:** Guojun Han, Zhaozhao Hu, Zhihui Zhao, Baoshun Zhu, Jianjun Chen

**Affiliations:** 1 College of Resources and Environment Sciences, Gansu Agricultural University, Lanzhou, China; 2 Pomology and Flower Research Institute, Gansu Academy of Agricultural Sciences, Lanzhou, China

**Keywords:** okra, synergistic effect, seed germination, salt stress, temperature gradient

## Abstract

**Introduction:**

Against the backdrop of global warming and intensifying soil salinization, the interactive effects of temperature and salt stress are reshaping physiological regulation during crop germination; however, their underlying synergistic mechanisms remain unclear. As a typical thermophilic crop, okra exhibits high sensitivity to thermal environments and water-salt fluctuations during seed germination, making it an ideal model for elucidating multi-factor stress response mechanisms.

**Methods:**

Through a two-factor controlled experiment comprising five temperature gradients (20-36°C) and three salt-induced composite stress intensity levels equivalent to -0.05, -0.1, and -0.2 MPa, this study systematically evaluated the coupled responses of germination traits and antioxidant systems in okra seeds.

**Results:**

The results demonstrate that temperature dominates germination metabolism, with 24-28°C identified as the optimal germination window. Conversely, the salt-induced composite stress acts as the primary limiting factor, with the -0.1 MPa equivalent intensity established as the critical physiological inflection point below which germination capacity is severely inhibited. Further analysis revealed that temperature significantly modulates the effects of salinity by regulating redox homeostasis, where optimal temperatures centered at 28°C alleviate cellular degradation, whereas high temperatures >32°C amplify salt-induced oxidative damage.

**Discussion and conclusion:**

Multivariate analysis elucidated a distinct metabolic cost and passive biomass loss mechanism. Under high temperatures, the combined constraint drives the accumulation of reactive oxygen species ($ROS$), manifested by increased MDA content. This severe physiological stress led to a profound cellular dysregulation accompanied by the upregulation of SOD, POD, and CAT activities, which represented an insufficient compensatory response that ultimately constrained germination potential. In conclusion, this study uncovers the core mechanism by which temperature modulates redox homeostasis to amplify salt stress effects. These findings provide novel empirical evidence for combined stress theories, offering a quantitative basis for the precise management of temperature and salinity in okra cultivation within saline-alkali regions.

## 1 Introduction

Soil salinization is a global land degradation threat that has affected over 25% of arable land worldwide, causing billions of dollars in economic losses annually (Wang et al., 2025). Climate change further exacerbates this issue through sea-level rise, prolonged droughts, and inappropriate soil and water management. Consequently, the land area affected by salinization continues to expand across all climatic zones and continents (Haj-Amor et al., 2022; Wang et al., 2025). Simultaneously, global warming exerts a direct negative impact on crop productivity: for every 1°C increase in average temperature, the yields of maize, wheat, and soybean decrease by approximately 7.5%, 6.0%, and 6.8%, respectively (Bernacchi et al., 2023). The compounding effects of salinization and climate warming impose unprecedented combined stress on global agricultural production and food security.

The seed germination stage is the most sensitive period to abiotic stress in the plant life cycle, and its efficiency directly determines the quality of crop population establishment and final yield (Munns and Tester, 2008; Hanin et al., 2016). Salt stress inhibits germination primarily through two pathways. First, it lowers the osmotic potential of the soil solution, inducing physiological drought and impeding seed water imbibition. Second, the excessive accumulation of toxic ions, such as Na^+^ and Cl^-^, disrupts intracellular ion homeostasis and key enzyme activities (Deinlein et al., 2014; Manono, 2025). Temperature, a core ecological factor regulating germination, directly determines germination vigor by influencing enzymatic reaction rates and hormonal balances. The warming trend and intensified temperature fluctuations can enhance seed dormancy and reduce germination percentages, jeopardizing field emergence uniformity and establishment success (Reed et al., 2022). Recent studies indicate that elevated temperatures amplify the stress effects of salinity on germination, and the combined stress of heat and salinity exerts an inhibitory effect that far exceeds the sum of the individual stresses (Khalid et al., 2023). Although this combined stress effect has been confirmed in certain model plants and staple crops, a systematic analysis of the physiological mechanisms underlying the synergistic regulation of temperature and salinity during the germination of specialty vegetable crops like okra is still lacking. Consequently, existing general principles are difficult to translate directly into species-specific, precise cultivation management strategies.

Okra, an annual thermophilic vegetable crop belonging to the Malvaceae family, is widely cultivated in warm regions of Asia and Africa and holds significant promotional value for the exploitation of marginal saline-alkali lands. However, okra seeds are highly sensitive to salinity during the germination stage; poor emergence and uneven stand establishment in saline-alkali soils constitute the core bottlenecks restricting its production potential (Naseem et al., 2023; Yang et al., 2025). Studies indicate that although okra exhibits relatively strong overall salt tolerance in moderate to severe saline-alkali soils, providing ecological benefits such as promoting soil desalination and improving soil fertility (Manono, 2025), the germination stage remains the weakest link in its salt tolerance profile. In contrast to model species and staple crops like Arabidopsis, rice, and wheat, where significant progress has been made in elucidating salt stress response mechanisms (Hu and Schmidhalter, 2023; Melino and Tester, 2023; Zhang et al., 2023), the physiological and molecular mechanisms of salt tolerance in okra remain largely unclear. Previous studies have predominantly focused on the independent effects of singular salinity or temperature factors. For instance, regarding salt stress, a concentration of 0.5% NaCl or higher can completely inhibit okra germination (Yang et al., 2025). Regarding thermal requirements, okra seeds demand relatively high temperatures for germination: the minimum soil temperature for emergence must exceed 15°C (Singh and Dhaliwal, 1972), and the optimal germination temperature range is 25–30°C (Amiri Monfared et al., 2017). Conversely, standalone high-temperature stress can significantly reduce its photosynthesis and fruit yield (Adelabu and Franke, 2022; Khan et al., 2022). Nevertheless, research evaluating the germination characteristics and antioxidant enzyme system responses of okra under the synergistic stress of temperature and salinity remains scarce, failing to provide adequate theoretical support for stress-resilient and precise cultivation in saline-alkali environments.

Given okra’s inherent thermotolerance and its potential for saline alkali land utilization, elucidating its response patterns to combined temperature and salt stress during germination is a fundamental prerequisite for understanding its early life history traits under changing environments. Therefore, this study employed okra seeds in a two factor completely randomized experiment, utilizing temperature gradients from 20 to 36 °C and osmotic potential levels induced by salt stress at -0.05, -0.1, and -0.2 MPa. We systematically quantified germination characteristics alongside the activities of superoxide dismutase, peroxidase, and catalase, as well as malondialdehyde content. The specific objective of this investigation is to determine how temperature modifies seed sensitivity to salt induced composite stress and to establish precise stress tolerance thresholds for this thermophilic species. Ultimately, these empirical data enrich the baseline theoretical framework regarding crop responses to combined abiotic stresses during early developmental stages, providing an essential foundation for future field based investigations.

## 2 Materials and methods

### 2.1 Plant materials and seed preparation

Seeds of the okra cultivar “*Luxing*” were used as the experimental material. These were purchased from the Henan Academy of Agricultural Sciences (Zhengzhou, China). According to the supplier’s certification, the germination percentage was ≥90% and purity was ≥98%. Prior to the experiment, seeds were surface-sterilized by soaking in a 1% (v/v) sodium hypochlorite (NaClO) solution for 10 min, followed by thorough rinsing with sterile deionized water 5~6 times to eliminate any residual disinfectant. After sterilization, surface moisture was blotted dry using sterile filter paper. The seeds were then placed in a desiccator at 4°C for 24 h to equilibrate and ensure uniform moisture content prior to the germination (Lv et al., 2021).

### 2.2 Experimental design and culture conditions

A two-factor completely randomized design was employed to evaluate the combined effects of temperature and salt stress. Five temperature regimens (20, 24, 28, 32, and 36°C)were established, as preliminary trials indicated an absence of germination at 16°C (Besma and Mounir, 2010). Among these, 28°C was designated as the physiological optimum reference temperature, aligning with the established optimal germination range of 25–30°C for okra (Ullah and Jan, 2024). Salt stress was simulated using sodium chloride (NaCl) solutions adjusted to three osmotic potentials: -0.05 MPa, -0.1 MPa, and -0.2 MPa (Saima et al., 2022), alongside a non-saline deionized water control (0 MPa; CK) (Yang et al., 2025). The precise NaCl concentrations required to achieve these potentials were calculated applying the Van ‘t Hoff equation (Michel and Kaufmann, 1973). All specific treatment combinations are summarized in **Table 1**.

**Table 1:** NaCl solution concentrations corresponding to different osmotic potentials at various temperatures.

Temperature°C Osmotic potential (MPa)	NaCl solution concentration(g/kg H_2_O)				
	20°C	24°C	28°C	32°C	36°C
CK	0	0	0	0	0
-0.05	0.601	0.593	0.585	0.577	0.570
-0.1	1.265	1.248	1.231	1.215	1.199
-0.2	2.670	2.634	2.599	2.565	2.532

The concentration of NaCl is expressed as grams of solute per kilogram of solvent (g/kg H_2_O) rather than per liter (g/L). This mass-based metric ensures the precise calculation of osmotic potentials across different temperature regimens, as it remains completely independent of temperature-induced volume fluctuations.The osmotic potential was calculated using the formula Ψs = -(1.18 × 10^-^²)C - (1.18 × 10^-4^)C² + (2.67 × 10^-4^)CT + (8.39 × 10^-7^)C²T (Michel and Kaufmann, 1973), where Ψs represents the osmotic potential (MPa) of the NaCl solution, C is the NaCl concentration (g/kg), and T is the temperature (°C).

Each treatment was replicated three times, with 50 seeds per replicate. Germination tests were conducted using the paper bed method, following the guidelines of the International Seed Testing Association (ISTA, 2022). Specifically, two layers of sterile filter paper were placed in 9-cm diameter Petri dishes. Each dish was moistened with 5 mL of the respective NaCl solution (or deionized water for the control), ensuring the filter paper was saturated without accumulation of free water. Seeds were arranged evenly to prevent overlapping. To minimize evaporation while maintaining gas exchange, the petri dishes were sealed with parafilm and covered with perforated plastic film (Guo et al., 2024).

The dishes were incubated in artificial climate chambers (MGC-3000H, Shanghai Yiheng Scientific Instrument Co., Ltd., China) set to the corresponding temperatures. To maintain constant osmotic potential and prevent salt accumulation, the treatment solutions were replaced, and the filter papers were rinsed with the respective solutions every 2 days. Seed germination and seedling growth were observed and recorded daily throughout the experiment. The cultivation conditions included a photoperiod consisting of 12 hours of light and 12 hours of darkness, a light intensity of 300 μmol·m^-2^·s^-1^, and a relative humidity of 70% ± 5%.

### 2.3 Measurement indices and methods

#### 2.3.1 Determination of seed germination indices

Germination was defined as the point when the radicle length reached half the length of the seed (Bai et al., 2020), with germination counts recorded at 12-hour intervals (Yang et al., 2025). Growth indices were evaluated on the ninth day. To account for stress-induced variations in emergence timing and to ensure an unbiased representation of the overall treatment effects, 15 seedlings were selected entirely at random from the total pool of successfully germinated seeds per treatment. This strictly randomized sampling strategy deliberately eliminated any potential selection bias toward the earliest-germinated or most vigorous individuals. Subsequently, radicle length was measured using a vernier caliper (0.01 mm precision), and seedling fresh weight was determined using an analytical balance (0.001 g precision) (Mohammadi and Amiri, 2010).

The germination indices were calculated using the following formulas:

Germination Percentage (GP):


$$\text{GP}=\frac{day\;9\;germination\;count}{total\;number\;of\;seeds\;tested}\times 100％$$

Germination Energy (GE):


$$\text{GE}=\frac{day\;4\;germination\;count}{total\;number\;of\;seeds\;tested}\times 100％$$

Germination Index (GI):


$$\text{GI}=\sum \frac{Gt}{Dt}$$

Vitality Index (VI):


VI=GI×S

(Bewley, 1997).

Where Gt is the number of seeds germinated on day t, Dt is the corresponding time in days, and S represents the fresh weight of seedlings on the 9th day. Biologically, these indices comprehensively quantify distinct aspects of seed performance under abiotic stress: Germination Percentage (GP) denotes the ultimate germination capacity of the seed population; Germination Energy (GE) measures the rapidity and uniformity of early germination, serving as a primary indicator of intrinsic seed vigor; the Germination Index (GI) evaluates the speed of germination, with higher values indicating faster germination; and the Vitality Index (VI) integrates germination speed with subsequent post-germination growth to holistically evaluate overall seedling establishment potential.

#### 2.3.2 Determination of biochemical indices

On day 10 of germination, fresh whole seedlings were sampled to determine the activities of antioxidant enzymes, including superoxide dismutase (SOD), catalase (CAT), and peroxidase (POD), as well as the malondialdehyde (MDA) content, a key indicator of cell membrane damage and permeability. Specifically, SOD activity was measured using the nitroblue tetrazolium (NBT) photochemical reduction method; CAT activity was determined via ultraviolet spectrophotometry; POD activity was assayed using the guaiacol colorimetric method; and MDA content was quantified utilizing the thiobarbituric acid (TBA) method (Li et al., 2023).

### 2.4 Statistical analysis

All experimental data were initially organized using Microsoft Excel 2021, and subsequent statistical analyses were performed using R software (version 4.5.1). A two-way analysis of variance (ANOVA) was employed to evaluate the effects of temperature, salt stress, and their interaction on the germination indices and physiological parameters of okra seeds. Multiple comparisons among treatment means were conducted using Fisher’s Least Significant Difference (LSD) test, with the significance level set at P < 0.05. Redundancy analysis (RDA) was performed using Canoco 5.0 software to explore the correlations between environmental factors (temperature and salt stress) and the germination as well as physiological indices. All graphs and charts were generated using Origin 2023 software (OriginLab Corp., MA, USA). All data are expressed as the mean ± standard deviation (SD).

## 3 Results

### 3.1 Variance analysis of environmental factors and their interactions

The results of the two-way analysis of variance confirmed that salinity, temperature, and their interaction exerted extremely significant effects on the morphogenesis, physiological metabolism, and biochemical indices of okra seeds (*P* < 0.05) (**Table 2**). Variance analysis revealed that salt stress accounted for a substantial proportion of the data variation, acting as the primary driving factor for most measured indices, with the exception of Germination Energy, which was more strongly driven by temperature. Crucially, the highly significant interaction between temperature and salt stress indicates that the physiological response of okra to salinity is profoundly contingent upon the thermal environment. This interaction emphasizes that temperature does not merely act as an independent variable but actively modulates the biochemical sensitivity of the seeds to salt stress. Consequently, while salt stress serves as the dominant constraint on the germination process, the specific temperature regime plays a critical role in either exacerbating or mitigating the salt-induced composite and oxidative damage.

**Table 2:** Effects of temperature, salt stress and their interaction on seed germination and antioxidant enzyme activity.

F	SOD	POD	CAT	MDA	GP	GE	GI	VI	RL	DW
T	438.6***	729.2***	1725.9***	2382.1***	43.8***	94.7***	78.8***	99.0***	102.9***	49.7***
S	1002.0***	1102.2***	4613.2***	2957.2***	58.6***	69.8***	69.3***	137.7***	122.1***	139.7***
T×S	11.3***	23.0***	104.3***	113.0***	3.3**	3.0**	1.0*	1.9*	3.7***	7.0***

The data in the table are F values of two-way ANOVA, * means there is significant influence at the 0.05 level, and ** indicates that there is significant influence at the 0.01 level. *** indicate significant effects at the p ≤ 0.001 level. T, Temperature; S, Osmotic potential; T×S, interaction between temperature and osmotic potential; GP, Germination Percentage; GE, Germination energy; GI, Germination index; VI, Vigor index; RL, Radicle length; DW, Seed dry weight; SOD, Superoxide dismutase; POD, Peroxidase; CAT, Catalase; MDA, Malondialdehyde.

### 3.2 Response of okra seed germination dynamics to temperature and salt stress

Growing Degree Days (GDD) were calculated to quantify the effective thermal time required for okra germination under varying levels of salt stress. Although previous literature suggests a general minimum thermal requirement of 15°C for okra (Singh and Dhaliwal, 1972), our specific preliminary trials demonstrated an absolute absence of germination at 16°C, which aligns with findings under similar experimental conditions (Besma and Mounir, 2010). Consequently, to most accurately reflect the physiological thermal threshold of the seeds utilized in this study, a base temperature (Tb) of 16°C was adopted. Given the constant temperature regimens employed, GDD was calculated using the following formula:


GDD= (Ti−Tb)×D

Where Ti represents the daily actual temperature, Tb is the base temperature (16°C), T is the constant temperature of the specific treatment regimen (20, 24, 28, 32, or 36°C), and D is the total number of days required to achieve germination.

**Figure 1:**
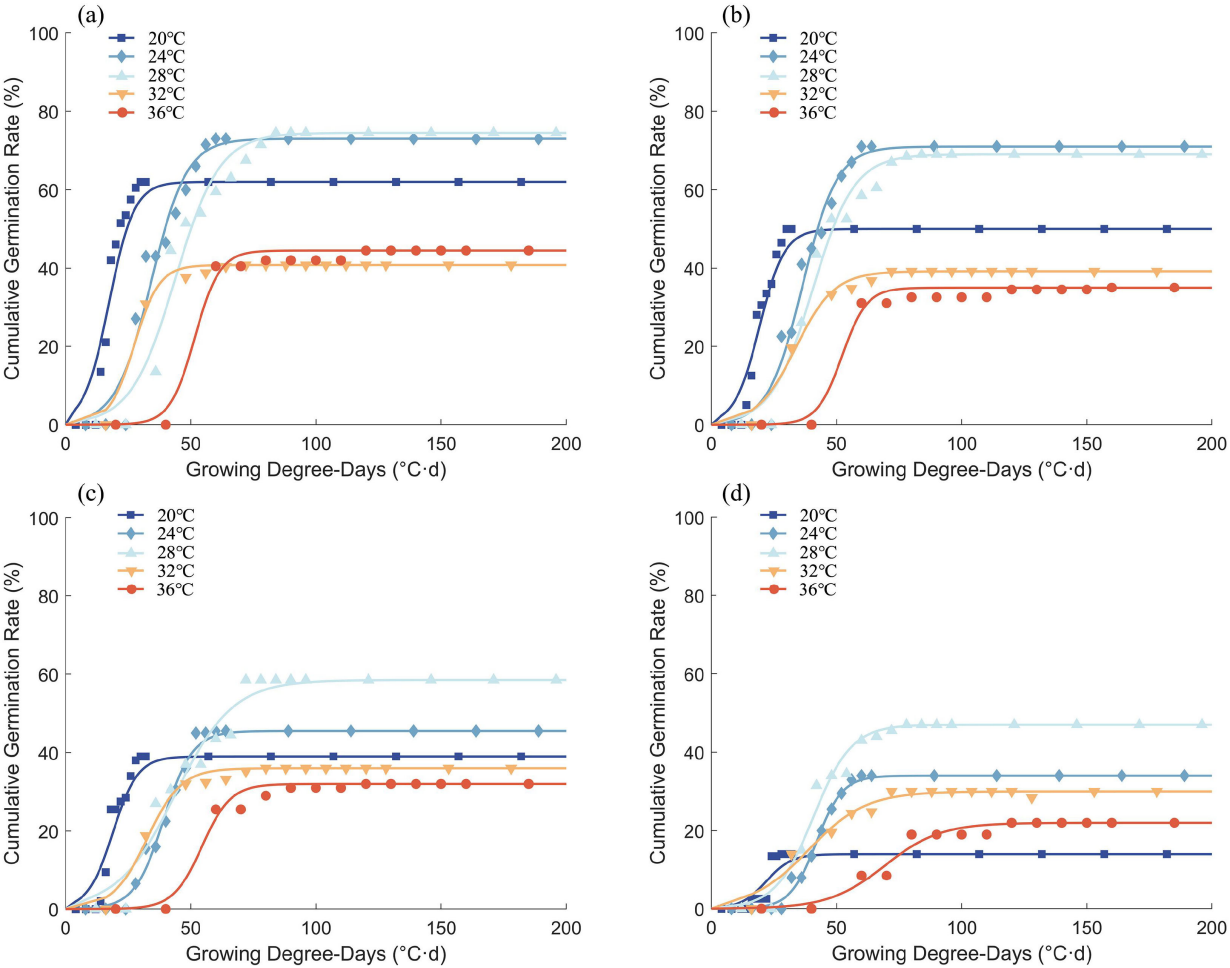
Dynamic response of okra seed germination percentage to accumulated temperature under different temperature and osmotic potential treatments. **(a)** Logistic fitted curves for okra seed germination percentage as a function of accumulated temperature under temperatures ranging from 20 to 36°C at an osmotic potential of 0 MPa. **(b)** Logistic fitted curves for okra seed germination percentage as a function of accumulated temperature under temperatures ranging from 20 to 36 °C at an osmotic potential of -0.05 MPa. **(c)** Logistic fitted curves for okra seed germination percentage as a function of accumulated temperature under temperatures ranging from 20 to 36°C at an osmotic potential of -0.1 MPa. **(d)** Logistic fitted curves for okra seed germination percentage as a function of accumulated temperature under temperatures ranging from 20 to 36°C at an osmotic potential of -0.2 MPa.

The interactive effect of temperature and salt stress significantly reduced the germination capacity of okra seeds and disrupted their developmental synchronization. As shown in **Figure 1**, kinetic analysis based on the Logistic model detailed these interactive responses, with all coefficients of determination R^2^ exceeding 0.94, indicating exceptionally high data reliability. The specific equation of the logistic model is presented in **Table 3**. The cumulative germination percentage followed a positive non-linear course with increasing GDD, displaying a characteristic sigmoidal trajectory defined by an initial lag phase, followed by a rapid exponential increase, and culminating in a stabilization plateau. Specifically, the optimal thermal window from 24 to 28 °C maximized the final germination potential, whereas salt stress exerted a strict concentration-dependent inhibitory effect, progressively reducing the germination capacity across all temperature gradients. Furthermore, temperature modulated the sensitivity of the seeds to salinity. The optimal temperature of 24 °C effectively buffered the damage of mild salt stress and maintained a high germination potential. Conversely, under the severe dual stress comprising an extreme high temperature of 36 °C and a heavy salinity of -0.2 MPa, the initial metabolic lag phase prior to germination was significantly prolonged, accompanied by an abrupt decline in both the intrinsic germination percentage and population uniformity.

**Table 3:** Coefficients of the logistic model for okra seed cumulative germination percentage as a function of GDD under different temperature and osmotic potential treatments.

T S	CK	-0.05MPa	-0.1MPa	-0.2MPa
20°C	K: 62.00 a: 2.5568 r: 0.1500	K: 50.00 a: 2.8717 r: 0.1500	K: 39.00 a: 2.8066 r: 0.1500	K: 14.00 a: 3.2857 r: 0.1500
24°C	K: 73.00 a: 4.8300 r: 0.1413	K: 71.00 a: 5.2905 r: 0.1453	K: 45.50 a: 5.8481 r: 0.1500	K: 34.00 a: 6.2783 r: 0.1500
28°C	K: 74.50 a: 4.9536 r: 0.1131	K: 69.00 a: 4.9022 r: 0.1211	K: 58.50 a: 3.7931 r: 0.0868	K: 47.00 a: 5.3483 r: 0.1312
32°C	K: 40.80 a: 4.1334 r: 0.1500	K: 39.20 a: 4.3384 r: 0.1287	K: 36.00 a: 4.7757 r: 0.1468	K: 30.00 a: 3.3667 r: 0.0870
36°C	K: 44.50 a: 7.7260 r: 0.1500	K: 35.00 a: 7.8091 r: 0.1500	K: 32.00 a: 8.1569 r: 0.1500	K: 22.00 a: 6.2144 r: 0.0898

K represents the maximum germination percentage of okra seeds; r represents the germination percentage parameter; a represents the germination initiation point.

### 3.3 Response of okra seed germination characteristics to temperature and salt concentration

**Figure 2:**
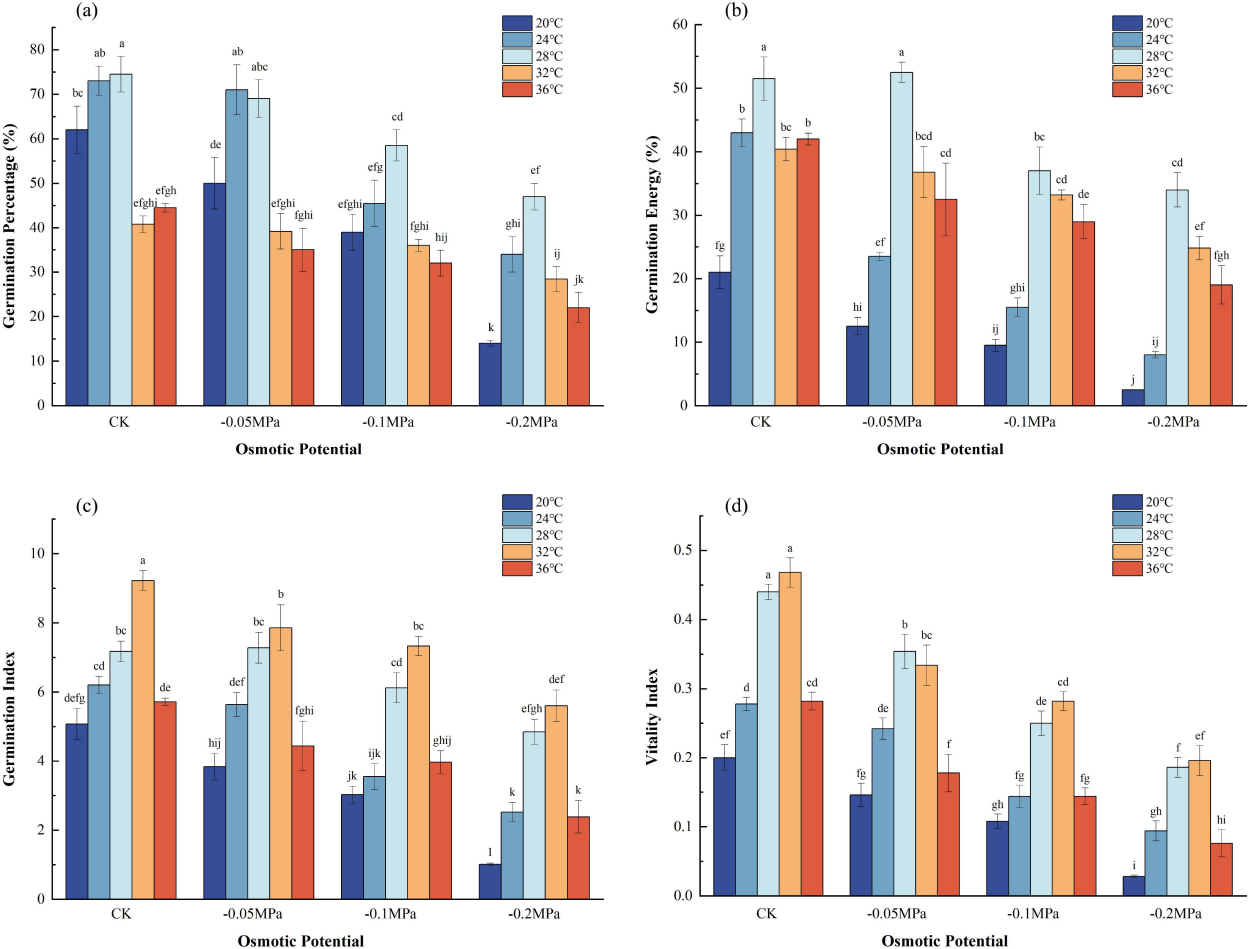
Differences in okra seed germination percentage, germination energy, germination index, and vigor index [**(a)** germination percentage; **(b)** germination energy; **(c)** germination index; **(d)** vigor index] under different osmotic potentials at various temperatures. Error bars represent standard errors. Within each subplot, the same letters indicate no significant difference between treatments (p > 0.05), while different letters indicate significant differences between treatments (p < 0.05); Osmotic potential treatments: 0 MPa (control, CK), -0.05 MPa, -0.1 MPa, and -0.2 MPa.

As illustrated in the **Figure 2**, temperature and osmotic potential exhibited a significant interactive effect on the germination characteristics of okra seeds (P < 0.05). Overall, as the osmotic potential decreased the germination percentage, germination energy, germination index, and vitality index of okra seeds all exhibited a significant downward trend. Conversely, at identical salinity levels, these indices displayed a distinct unimodal response pattern with increasing temperature, characterized by an initial rise followed by a subsequent decline. Specifically, the germination percentage and germination energy reached their peaks at 28 °C. Notably, at this optimal temperature of 28 °C, the mild NaCl-induced osmotic potential of -0.05 MPa did not exert a significant inhibitory effect on germination percentage and germination energy, resulting in performance statistically comparable to the non-stressed control. In contrast, under this identical mild stress, the 20 °C and 32 °C treatment groups already experienced significant reductions. Furthermore, although the germination index and vitality index attained their maximum values at 32 °C, these figures were not statistically significantly different from those observed in the 28 °C treatment group. As the salinity intensified to the severe osmotic potential level of -0.2 MPa, the reductions in all germination indices reached their maximum across all temperature groups. Among these, the 20 °C sub-optimal temperature group proved to be the most sensitive to this severe constraint, with all indices plummeting by 77.41% to 88.10% relative to their corresponding controls. In contrast, the baseline germination vitality in the 28 °C treatment group was optimally preserved even under this severe constraint. The declines in its germination percentage, germination energy, germination index, and vitality index relative to the corresponding control were comparatively moderate, standing at 36.91%, 33.98%, 32.37%, and 57.72%, respectively.

### 3.4 Response of okra seedling growth to temperature and salt stress

**Figure 3:**
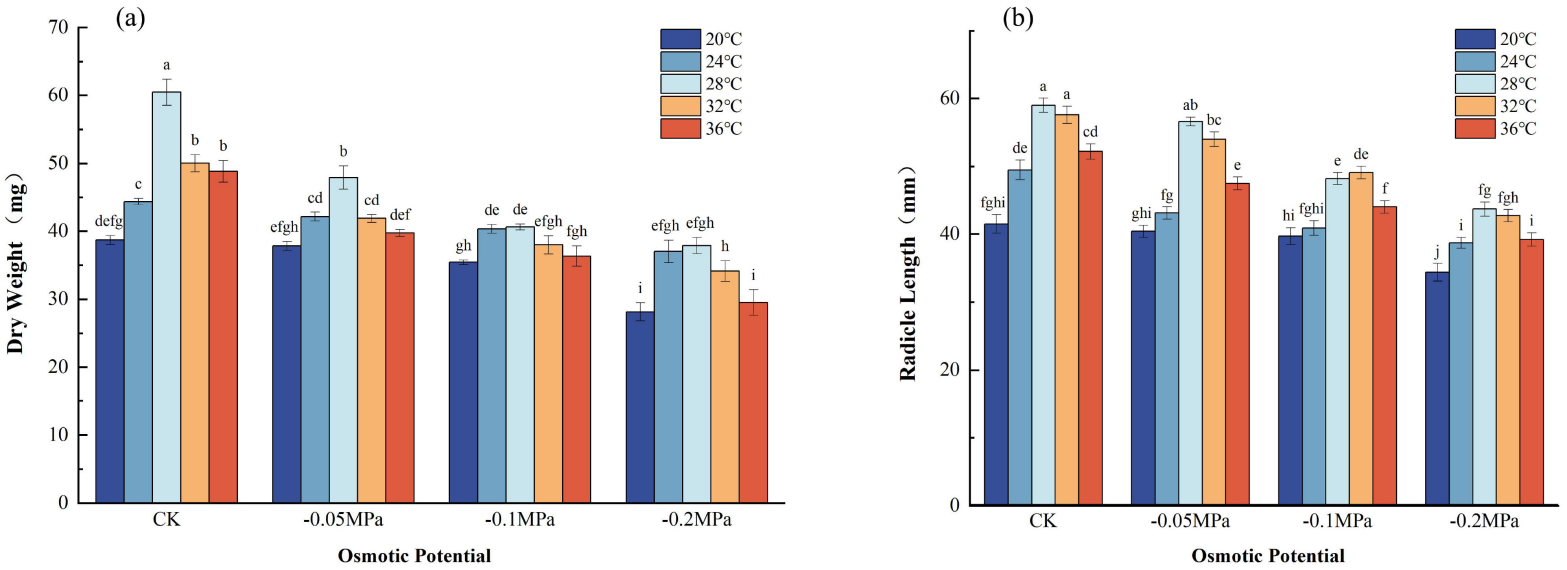
Differences in okra seed dry weight and radicle length [**(a)** dry weight; **(b)** radicle length] under different osmotic potentials at various temperatures. Bars represent standard errors. Within each subplot, the same letters indicate no significant difference between treatments (p > 0.05), while different letters indicate significant differences between treatments (p < 0.05); Osmotic potential treatments: 0 MPa (control, CK), -0.05 MPa, -0.1 MPa, and -0.2 MPa.

As shown in **Figure 3**, the interaction between temperature and osmotic potential significantly affected the morphogenesis and biomass accumulation of okra seedlings (*P* < 0.05). Overall, as osmotic potential decreased, both radicle length and seedling dry weight exhibited a significant downward trend. However, under the same osmotic potential, these metrics displayed a trend of initially increasing and then decreasing with rising temperature.

Among the different stress intensities, the -0.2 MPa treatment caused the most severe inhibition of seedling growth. Compared to the control (CK), the reductions in radicle length and seedling dry weight across all temperatures ranged from 17.17%-25.90% and 16.73%-39.56%, respectively. However, temperature demonstrated a significant regulatory effect:28 °C degrees Celsius not only was the optimal condition for growth but also exhibited a stress mitigation effect. Specifically, under a mild stress level with an osmotic potential of negative 0.05 MPa, the radicle length in the 28°C group showed no significant difference compared to the CK group. However, the radicle dry weight under this condition was lower than that of the CK. This indicates that although okra seedlings maintain root elongation at this temperature, the mild salt stress alters the radicle structure, making it thinner.

### 3.5 Response of okra seedling MDA content to temperature and salt stress

**Figure 4:**
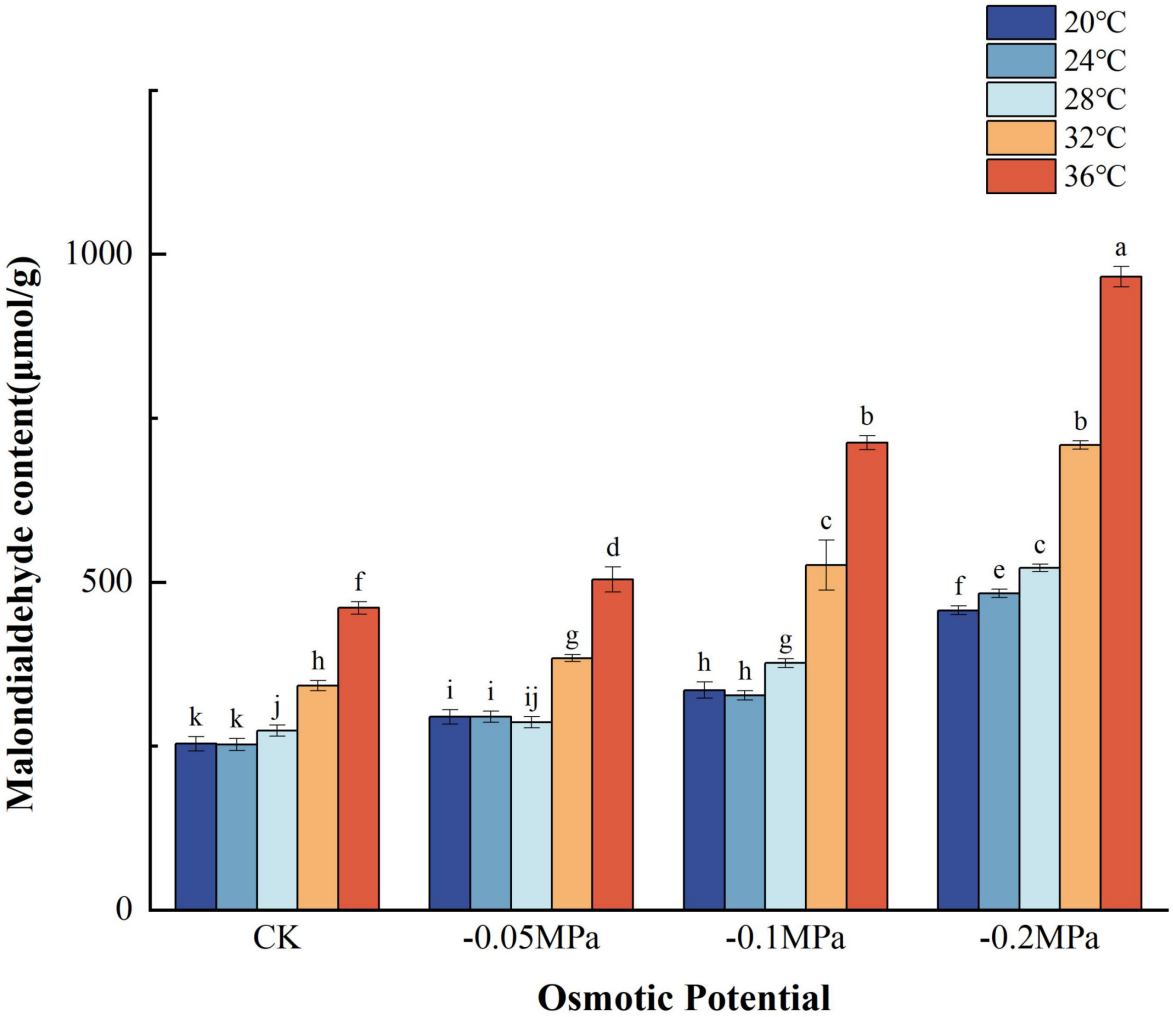
Comparison of malondialdehyde (MDA) content in okra seeds under different osmotic potentials at various temperatures [**(a)** 20°C; **(b)** 24°C; **(c)** 28°C; **(d)** 32°C; **(e)** 36°C]. Bars represent standard errors. Within each subplot, the same letters indicate no significant difference between treatments (p > 0.05), while different letters indicate significant differences between treatments (p < 0.05). Osmotic potential treatments: 0 MPa (control, CK), -0.05 MPa, -0.1 MPa, and -0.2 MPa.

As shown in **Figure 4**, the interaction between temperature and salt stress significantly influenced MDA accumulation in okra seedlings (*P* < 0.05). Generally, as osmotic potential decreased (salt stress intensified), MDA content increased significantly across all temperature treatment groups. However, the accumulation patterns varied significantly under different stress intensities. Under mild stress (-0.05 MPa), MDA content in the low and optimal temperature zones (20~28°C) showed no significant increase compared to the control (CK), suggesting relatively minor cell membrane damage. In contrast, in the high temperature zones (32°C and 36°C), even mild stress caused a significant surge in MDA content, implying that high temperature exacerbated the oxidative damage induced by salt stress. When the salt stress intensity reached the NaCl-induced osmotic potential level of -0.2 MPa, MDA content peaked in all temperature groups, with increases ranging from 80.29% to 109.67% relative to CK, indicating that the cell membranes underwent severe oxidative peroxidation at this stage. Generally, the lipid peroxidation of okra seeds remains relatively constrained under non-stressed control and mild osmotic potential conditions. However, when the NaCl-induced stress intensity falls below the -0.1 MPa osmotic threshold, the rate of lipid peroxidation accelerates sharply, and supra-optimal temperature environments further amplify the physiological damage caused by this severe combined adversity.

### 3.6 Response of okra seedling antioxidant enzyme activities to temperature and salt stress

**Figure 5:**
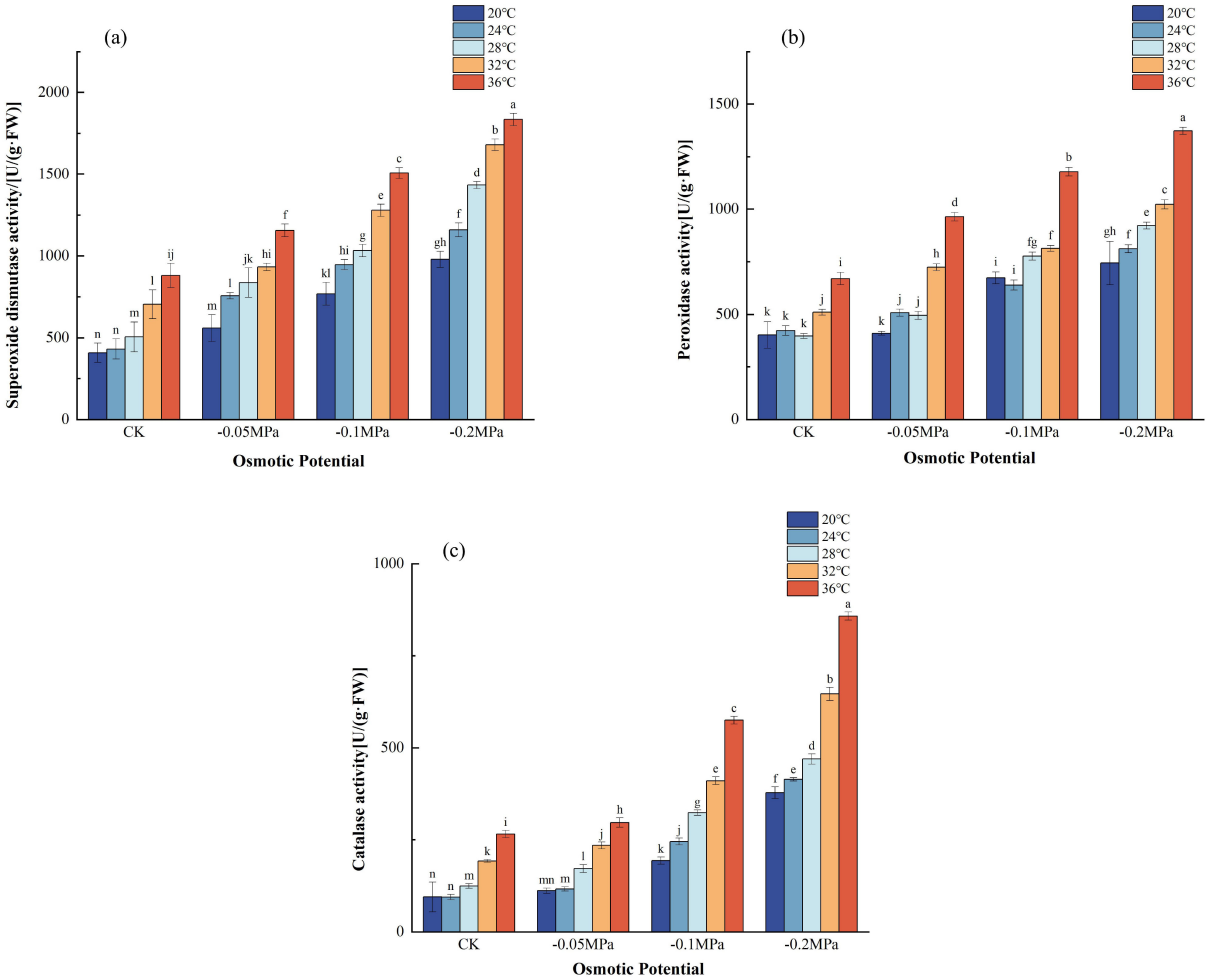
Differences in superoxide dismutase, peroxidase, and catalase activities [**(a)** superoxide dismutase; **(b)** peroxidase; **(c)** catalase] in okra seeds under different osmotic potentials at various temperatures. Error bars represent standard errors. Within each subplot, the same letters indicate no significant difference between treatments (p > 0.05), while different letters indicate significant differences between treatments (p < 0.05). Osmotic potential treatments: 0 MPa (control, CK), -0.05 MPa, -0.1 MPa, and -0.2 MPa.

As shown in **Figure 5**, in terms of the overall trend, as the NaCl-induced osmotic potential of the medium decreased, the activities of all three antioxidant enzymes (SOD, POD, and CAT) exhibited a significant dose-dependent increasing trend. Specifically, under mild stress with an osmotic potential of -0.05 MPa, the rise in enzyme activity was moderate. Notably, within the sub-optimal to optimal temperature range of 20-28 °C, SOD and POD activities showed only minor fluctuations, remaining statistically comparable to the basal levels of the control (CK). However, under severe constraint equivalent to the -0.2 MPa osmotic threshold, the antioxidant enzyme activities were maximally induced. Compared to CK, SOD and POD activities were significantly upregulated, increasing by 108.27-183.29% and 84.91-131.93%, respectively. CAT showed the most sensitive and drastic response to salinity; its activity climbed sharply as the salt stress intensified, reaching the maximum observed values across all temperature groups at the severe osmotic potential level of -0.2 MPa, which represented a significant increase of 223.32-338.84% compared to CK. Furthermore, temperature exerted a significant modifying effect on this compensatory response. While the induction amplitude of enzyme activity remained relatively stable across the 20-28 °C thermal range, the application of supra-optimal high temperatures (32 °C and 36 °C) combined with high salinity created a robust superposition effect, resulting in an explosive, compensatory upregulation in the activities of all three enzymes as the osmotic potential decreased.

### 3.7 Correlation analysis between germination indices and antioxidant enzyme activities

**Figure 6:**
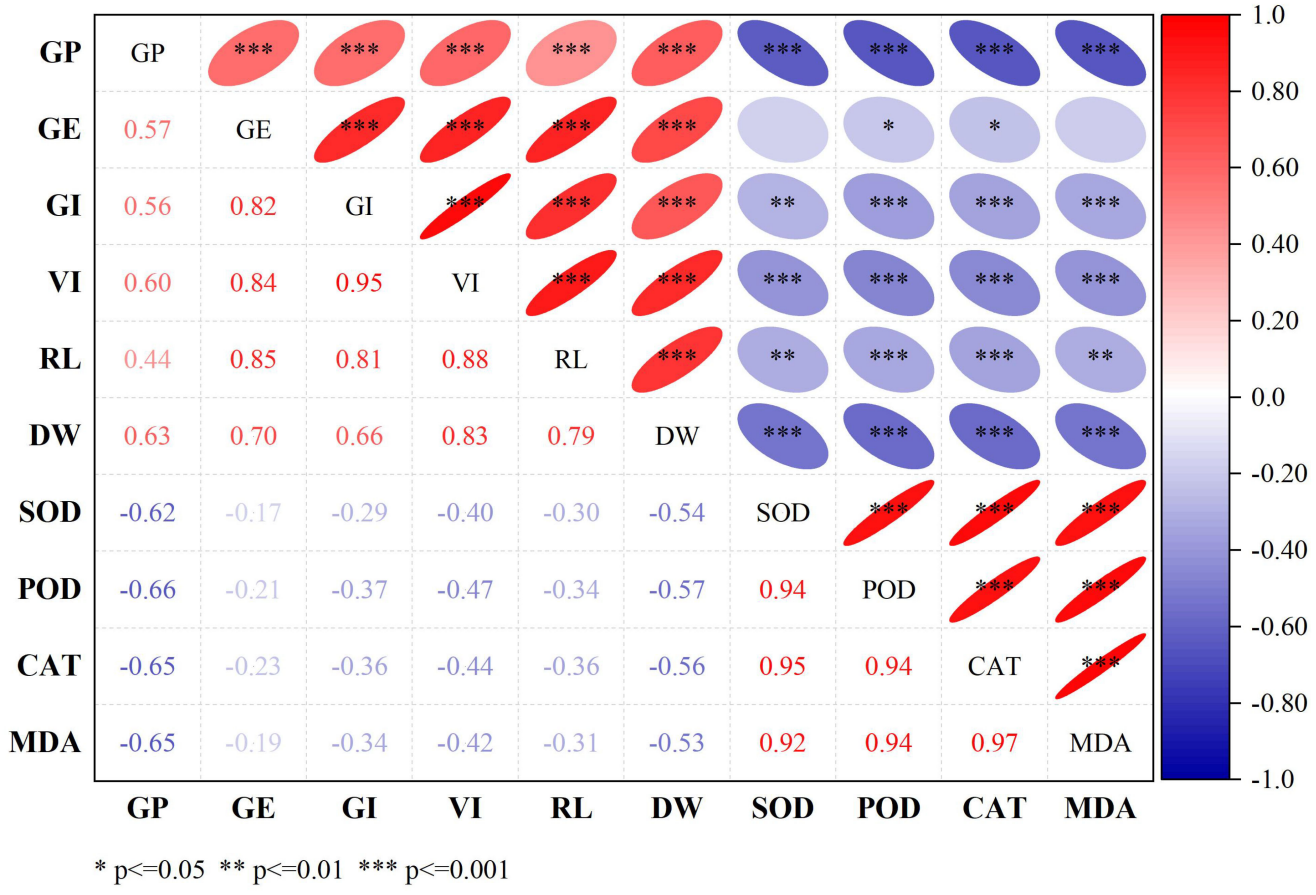
Pearson correlation matrix among germination traits and physiological indicators of okra seeds under temperature and salt stress. Asterisks *, **, and *** indicate significance at *p* < 0.05, *p* < 0.01, and *p* < 0.001, respectively. GP, germination percentage; GE, germination energy; GI, germination index; VI, vitality index; RL, radicle length; DW, seed dry weight; SOD, superoxide dismutase; POD, peroxidase; CAT, catalase; MDA, malondialdehyde.

Under combined temperature and salinity stress, the germination process of okra seeds exhibits a pronounced physiological constraint driven by cellular dysregulation, indicating that the insufficient compensatory upregulation of the antioxidant system reflects a severe disruption of germination potential and growth quality. As shown in **Figure 6**, Pearson correlation analysis quantitatively confirmed this metabolic disruption process. The malondialdehyde content, which signifies the extent of cell membrane damage, demonstrated an extremely significant synchronous accumulation trend with the activities of these enzymes, including superoxide dismutase, peroxidase, and catalase (r:0.92-0.97, p ≤ 0.001). Simultaneously, malondialdehyde exerted a consistent and extremely significant negative constraint on morphological developmental phenotypes, such as the final germination percentage (r = -0.62, p ≤ 0.001) and seedling dry weight (r = -0.53, p ≤ 0.001). This definitive antagonistic pattern demonstrates that the severe oxidative damage induced by the combined environmental stressors causes a profound systemic disorder, where the non-adaptive surge in enzymatic activities accompanies a drastic passive reduction in biomass synthesis. Consequently, this metabolic disruption constitutes the core physiological trajectory through which environmental stress restricts the early development of okra.

### 3.8 Redundancy analysis of environmental factors and physiological responses

**Figure 7:**
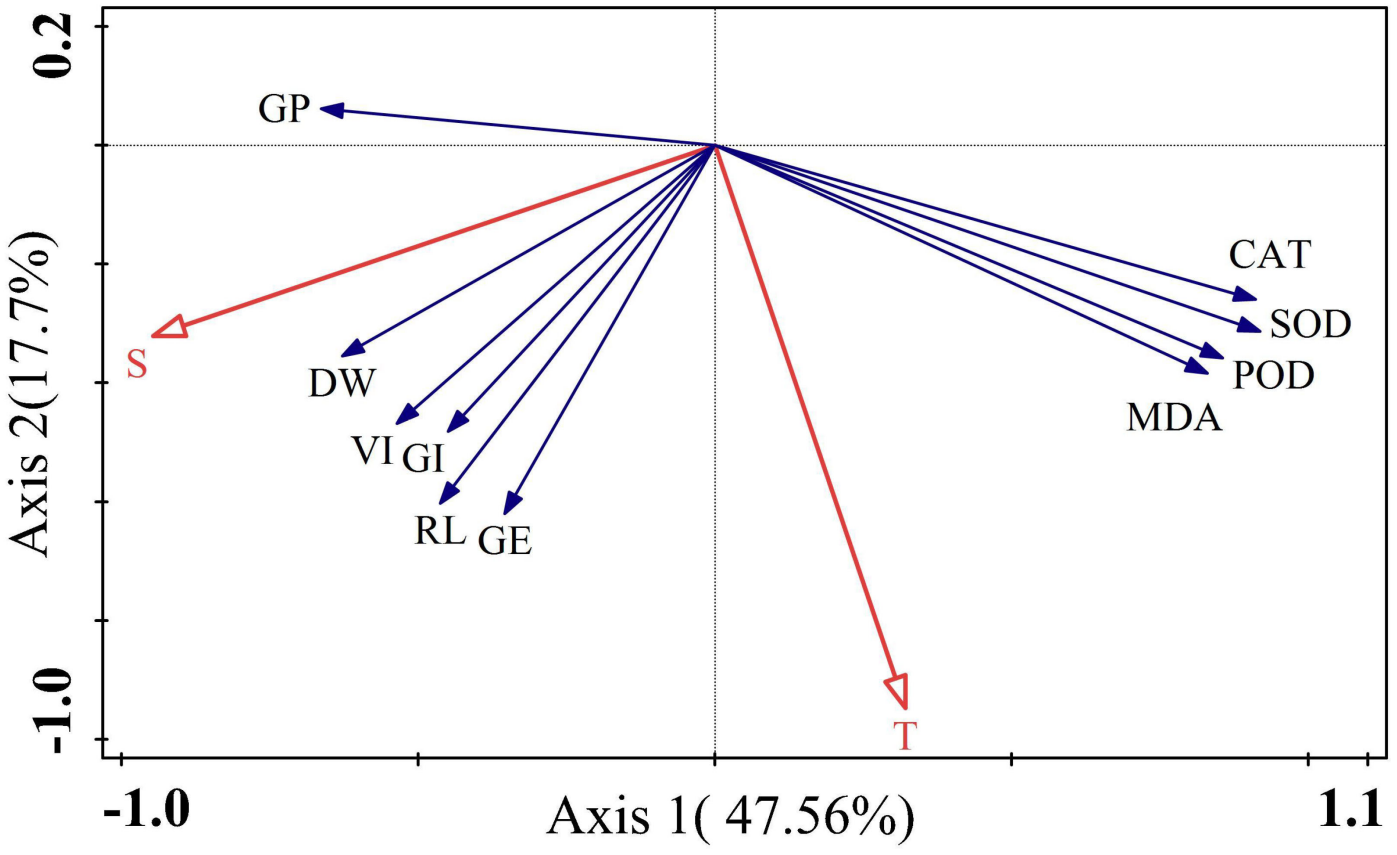
Redundancy analysis (RDA) of temperature and osmotic potential with germination and physiological indicators in okra seeds. T, Temperature; S, Osmotic potential; GP, Germination percentage; GE, Germination energy; GI, Germination index; VI, vitality index; RL, Radicle length; DW, Seed dry weight; SOD, Superoxide dismutase; POD, Peroxidase; CAT, Catalase; MDA, Malondialdehyde.

RDA was utilized to reveal the multivariate correlation patterns among environmental factors including T and S, germination traits including GP, GE, GI, VI, RL, and FW, and physiological response variables including SOD, POD, CAT, and MDA. As shown in **Figure 7**, the results showed that the first two canonical axes cumulatively explained 65.26% of the constrained variance in the response variables. Specifically, Axis 1 accounted for 47.56% of the variance, and Axis 2 accounted for 17.7%, indicating that this ordination model effectively reflects the driving effects of combined environmental factors on the response mechanisms of okra.

Regarding the vector distribution, the environmental vectors representing T and S exhibited a perpendicular relationship, indicating that their main effects in the overall ordination model were relatively independent. The developmental indicator vectors, namely DW, VI, GI, RL, and GE, were densely distributed between the T and S vectors and formed distinct acute angles with the S vector. Since S is characterized by negative values, this positive numerical correlation reflects a synchronous variation trend between the water potential level and both seedling growth quality and population vitality. Furthermore, the GP vector pointed to the upper left, forming an acute angle with the S vector and an obtuse angle with the T vector. This geometric feature signifies the negative driving effect of high temperature on GP.

At the physiological response level, SOD, POD, CAT, and MDA were highly clustered and pointed to the lower right, forming acute angles with the codirectional T vector. If the S vector is rotated by 180 degrees to represent the actual increase in salt stress concentration, the angles between these physiological stress indicator vectors and this reverse extension line become smaller than their angles with the T vector. This geometric distribution quantitatively demonstrates that, compared to the main effect of T, the increase in salinity exerts a more significant driving effect on the cell membrane lipid peroxidation level and the activity of the antioxidant defense system.

### 3.9 Summary of the regulatory mechanism

**Figure 8:**
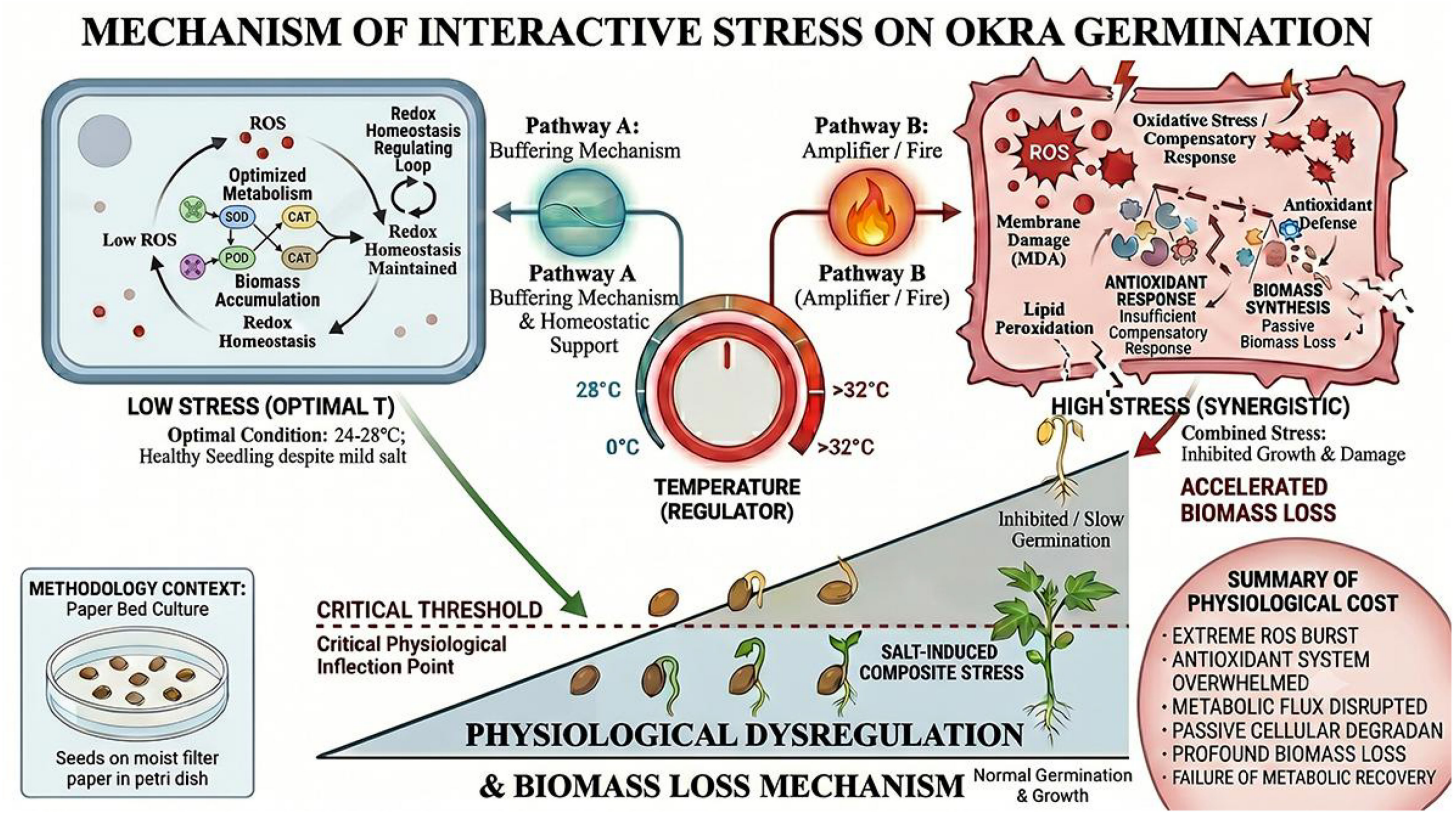
This image illustrates how temperature, acting as a key regulatory factor, synergistically modulates the germination process and antioxidant responses of okra seeds in conjunction with osmotic stress. The diagram highlights the buffering or shielding mechanism of seeds at the optimal temperature of 28 degrees Celsius, which maintains low reactive oxygen species levels and enzyme homeostasis to enable normal germination under mild salt stress. Conversely, it depicts the mechanism by which high temperatures above 32 degrees Celsius act as a stress signal amplifier, exacerbating the salt stress induced reactive oxygen species burst and triggering lipid peroxidation and membrane damage. Furthermore, the image reveals the reactive oxygen species mediated signaling pathways, designated as Pathway A and Pathway B, the core role of the antioxidant defense system in maintaining cellular homeostasis, and the resource allocation involving metabolic costs and passive biomass loss. It clarifies the critical threshold for the synergistic action of temperature and osmotic potential, thereby elucidating the physiological mechanisms underlying the inhibition of seed germination under combined stress. The abbreviations are defined as SOD for superoxide dismutase, POD for peroxidase, CAT for catalase, MDA for malondialdehyde, and ROS for reactive oxygen species.

The comprehensive synergistic regulatory mechanism is illustrated below (**Figure 8**).

## 4 Discussion

### 4.1 Metabolic regulatory mechanisms of temperature on okra seed germination

Temperature, as a pivotal ecological factor governing plant life activities, controls the processes of material metabolism and energy conversion within seeds primarily by regulating enzyme activity. Existing theories suggest that appropriate temperature increases significantly enhance the activities of germination-related enzymes, accelerating the hydrolysis and transport of stored reserves; however, exceeding critical thresholds leads to the denaturation and inactivation of enzyme proteins (Cherrate et al., 2023).

This study confirmed, through the quantification of germination indices and kinetic parameters, that temperature significantly reshaped the germination kinetics of okra seeds. The germination energy (GE), germination index (GI), and vitality index (VI) did not increase monotonically with temperature but instead exhibited a typical unimodal pattern, peaking at 28 °C. This is highly consistent with the maximum germination potential peaking within the 24-28 °C optimal thermal range. These results indicate that this range represents the optimal thermodynamic window for okra seeds to break dormancy, achieve efficient energy conversion, and undergo rapid embryonic axis elongation. In contrast, while the temperature of 20 °C did not delay the initiation of the metabolic process in terms of thermal time, it strictly limited the absolute germination capacity and rendered the seeds extremely vulnerable to severe salinity. Crucially, the kinetic analysis revealed that extreme high temperatures centered at 36 °C did not accelerate early metabolism but rather significantly prolonged the metabolic lag phase. The exacerbated oxidative damage at these supraoptimal temperatures ultimately led to a significant decline in overall seedling vigor and germination uniformity. Specifically, this suppression of vigor was manifested as a typical passive biomass loss along with the inhibition of root and shoot elongation. These findings align with those of Toscano and Saha in drought stress studies, which demonstrated that severe water deficits similarly restrict these crucial early growth parameters (Toscano et al., 2017; Saha et al., 2022).

This superior performance centered at 28 °C strongly confirms the “thermophilic nature” of okra, which is highly consistent with previous findings identifying 25-30 °C as the optimal germination range for this species (Ullah and Jan, 2024). Furthermore, preliminary trials and the data from this study collectively revealed the sensitivity of okra to extreme temperatures: from metabolic stagnation (cold dormancy) caused by low temperatures to irreversible thermal injury induced by super-high temperatures. This complete “bell-shaped” response pattern reaffirms the biological law that the temperature regulation of seed germination strictly follows the “three cardinal temperatures” principle.

### 4.2 Osmotic stress and threshold effects of salt stress on germination characteristics

Seed germination represents the most salt-sensitive window in the plant life cycle. Salt stress inhibits germination primarily by lowering environmental osmotic potential to induce “physiological drought,” restricting seed imbibition. This is accompanied by ion toxicity, which disrupts the basic physiological homeostasis and triggers complex, yet insufficient, passive compensatory behaviors within cellular metabolic pathways (Wang et al., 2023).

To quantitatively deconstruct these water-salt interactions, our controlled experiment utilized a strategic gradient of NaCl-induced osmotic potentials (-0.05, -0.1, and -0.2 MPa). These selected hydrostatic parameters provide a critical mechanistic link to real-world agricultural scenarios. Specifically, the mild salt stress equivalent to -0.05 MPa closely correlates with the rhizosphere soil solution water potential typically encountered during the spring sowing season in mildly salinized or adequately drained alluvial soils. Conversely, the drastic drops to the -0.1 MPa and -0.2 MPa thresholds accurately simulate the severe multi-factor water-salt constraints generated in the crop root zone during periods of intense topsoil evaporation and surface crusting in typical arid and semi-arid saline-alkali regions. Consequently, elucidating seed responses under these specific gradients bridges the gap between controlled hydrostatic experiments and field-scale agronomic applications.

This study elucidated a significant threshold effect in the response of okra seeds to salt stress. In preliminary trials, a severe salt stress intensity equivalent to -0.4 MPa resulted in complete germination failure, indicating that this stress level disrupted the seeds’ basic osmotic and chemical homeostasis (Saima et al., 2022). In the formal experiment, as the simulated salt-induced stress intensity intensified, the germination percentage (GP), germination energy (GE), and vitality index (VI) all exhibited downward trends. This aligns with the conclusions of Liang (Liang et al., 2018), who reported that salinity delays germination and reduces the final germination percentage by exerting combined osmotic and ionic constraints. However, the physiological response exhibited distinct phasic differences: mild stress (-0.05 MPa) had a negligible impact on all germination indices, suggesting that this low concentration constraint did not exceed the baseline physiological buffering capacity of okra seeds. Yet, once the salt-induced composite stress intensity exceeded the -0.1 MPa threshold, all indices experienced a precipitous decline. This profound germination inhibition under severe combined stress is consistent with findings related to water and ionic limitations (Yigit et al., 2016; Channaoui et al., 2019). Specifically, a low external water and chemical potential critically hinders initial seed hydration and induces toxic ions accumulation, thereby inhibiting the hydrolysis of stored carbohydrates and their subsequent transport to the embryonic axis. Consequently, this metabolic and energy deficit impedes the radicle from breaching the seed coat, ultimately leading to delayed emergence and significantly reduced seedling vigor.

Redundancy Analysis and correlation analysis indicated that salt-induced composite stress is the primary environmental screening factor restricting okra germination. The value of -0.1 MPa can be regarded as the critical physiological inflection point for okra seed response under this combined adversity. When the stress intensity falls below this threshold, singular osmotic adjustment is insufficient to maintain cell turgor pressure. Consequently, the severe accumulation of reactive oxygen species triggers a profound cellular dysregulation accompanied by the upregulation of antioxidant enzyme activities. This physiological constraint results in a typical metabolic costs and passive biomass loss, which is ultimately manifested as a significant loss in germination potential and seedling biomass.

### 4.3 Effects of temperature and salt stress on the seed lipid peroxidation and antioxidant enzyme activities

Secondary oxidative stress induced by salinity is a major barrier limiting germination. Excessive production of reactive oxygen species (ROS) is a typical physiological response to such abiotic stress. Specifically, ROS products, including superoxide anions and hydroxyl radicals, fiercely attack biological membrane systems and induce severe lipid peroxidation. Consequently, malondialdehyde (MDA) is widely regarded as a key indicator of the degree of cell membrane damage (Lotfi et al., 2019). Consistent with this mechanism, as the NaCl-induced osmotic potential decreased in our treatments, the potential oxidative stress attacked membrane lipid unsaturated fatty acids, leading to massive MDA accumulation and disrupted membrane integrity (Wang et al., 2001; Gao et al., 2003). In this study, MDA content peaked under the severe -0.2 MPa composite treatment, coinciding highly with the inhibition trend of germination traits. This suggests that membrane lipid peroxidation reflects the severe intensity of environmental constraints and serves as a key physiological cause of germination inhibition, aligning with the conclusions of Biswas that water and salt deficits lead to elevated MDA concentrations in seedlings (Biswas et al., 2023).

Temperature significantly modulated the seed’s physiological response patterns to this oxidative pressure. At the optimal temperature of 28 °C, the cellular damage remained minimized under the mild salt stress of -0.05 MPa, indicating that this mild constraint had not yet exceeded the baseline buffering capacity of the germinating seeds, and basal enzyme levels remained sufficient to maintain basic metabolic demands. However, high temperatures (> 32 °C) formed a malignant synergy with salt stress. This “Heat-Salt” superposition effect amplified cellular toxicity and oxidative damage, forcing the seed into a state of metabolic costs and passive biomass loss. Interestingly, under these supra-optimal temperatures (> 32 °C), the application of mild salinity (-0.05 MPa) did not trigger an immediate linear collapse in germination phenotypes, superficially resembling a “cross-adaptation” or priming-like trend. Nevertheless, the absence of broader kinetic data constrains any definitive attribution to an active, successful evolutionary cross-tolerance strategy. This apparent trend instead reflects a temporary baseline delay within the seed’s rigid physiological buffering boundaries, where the minor ionic and osmotic disturbance of -0.05 MPa has not yet entirely overwhelmed the residual homeostatic framework. Once the environmental adversity intensifies beyond this minor threshold, this passive buffering capacity is completely obliterated. Generally, plants exhibit complex antioxidant enzyme variations when subjected to oxidative stress induced by abiotic adversities (Zhang et al., 2015; Bai et al., 2018). Among these components, enzymes such as superoxide dismutase (SOD), peroxidase (POD), and catalase (CAT) are critical markers associated with cellular redox status, and their activities reflect the extent of disturbance within the internal metabolic system (Ahmad et al., 2008; Gill and Tuteja, 2010; Isabella et al., 2023). Consistent with this response framework, our results showed that the non-adaptive surge in enzymatic activities accompanied a drastic passive reduction in biomass synthesis, leading to the significant upregulation of SOD, POD, and CAT activities. Crucially, this pattern of enzyme activation aligns highly with the previously described MDA accumulation trend, suggesting a profound metabolic disruption where severe lipid peroxidation accompanies a enzymatic upregulation (Sen and Puthur, 2020). This strongly demonstrates that under the combined adversity of heat and severe salinity, germinating seeds undergo a severe cellular dysregulation, leading to an insufficient compensatory response manifested by extremely high levels of enzyme activity under exacerbated oxidative injury.

This acute stress response stands in sharp contrast to the baseline physiological status observed under optimal environmental conditions. Although the compensatory upregulation of the defense enzymes occurred to a significant extent, it accompanied a severe depletion of the energy reserves required for germination without effectively preventing membrane lipid peroxidation. Thus, high temperature effectively lowered the physiological critical inflection point of okra seeds under salinity constraints; mitigating heat stress is a prerequisite for preserving its baseline germination potential and ensuring seedling quality in complex habitats. Although this study has successfully characterized the synchronous fluctuations between antioxidant enzyme activities and lipid peroxidation, certain methodological boundaries remain to be addressed. The lack of direct upstream reactive oxygen species (ROS) kinetic measurements (e.g., H_2_O_2_ quantification), absolute ion homeostasis status (Na^+^/K^+^ ratios), and internal osmolyte profiles (e.g., proline and soluble sugars accumulation) limits our ability to make definitive causal inferences regarding alternative physiological defense pathways. Therefore, these unquantified multi-dimensional parameters represent crucial trajectories that require further comprehensive exploration in the future using metabolomics, ionomics, or molecular biology approaches to enrich the holistic theoretical framework of crop germination under interactive abiotic stresses.

## 5 Conclusion

Through a two-factor experiment involving temperature and salt stress, this study systematically elucidated the coupled and synergistic effects of temperature gradients and salinity on the germination and physiological metabolism of okra seeds. The research identified 24-28 °C as the optimal thermal window for okra under salt adversity, within which cellular damage remained minimized and the baseline germination vitality was optimally preserved. Simultaneously, -0.1 MPa was defined as the critical physiological inflection point for okra under combined stress; once the salt-induced composite stress intensity exceeded this threshold, the explosive accumulation of lipid peroxidation products resulted in severe and irreversible cellular dysregulation.

Crucially, temperature significantly modified the seed’s physiological response patterns to salt stress. Optimal temperatures effectively buffered mild salt stress, whereas high temperatures > 32 °C combined with severe salinity formed a malignant synergistic effect that significantly amplified oxidative damage. This interactive stress substantially heightened the metabolic costs and accelerated the passive biomass loss, where the upregulation of antioxidant enzyme activities reflected a severe cellular dysregulation that occurred at the severe expense of germination potential. While these controlled laboratory findings offer highly informative physiological insights for agronomic practice, translating these hydrostatic outcomes into field-scale recommendations requires appropriate caution. Based on our controlled hydro-thermal simulations, optimizing the sowing period within a thermal window of 24-28 °C and maintaining the equivalent field rhizospheric potential above -0.1 MPa through precise irrigation, drainage, or soil amendments represent a theoretically viable mitigation pathway to circumvent the malignant “Heat-Salt” synergy. Nevertheless, because real-world soil ecosystems exhibit dynamic multi-factor fluctuations, these laboratory-derived guidelines should be cautiously interpreted as foundational ecological baselines. Future field-scale agronomical trials are fundamentally required to validate and recalibrate these specific management thresholds in complex, open agricultural habitats before broad-scale field implementation.

## Data Availability

The original contributions presented in the study are included in the article/supplementary material. Further inquiries can be directed to the corresponding author.
